# The intestinal microbiome, weight, and metabolic changes in women treated by adjuvant chemotherapy for breast and gynecological malignancies

**DOI:** 10.1186/s12916-020-01751-2

**Published:** 2020-10-21

**Authors:** Atara Uzan-Yulzari, Maya Morr, Hala Tareef-Nabwani, Oren Ziv, Dafna Magid-Neriya, Ran Armoni, Efrat Muller, Anca Leibovici, Elhanan Borenstein, Yoram Louzoun, Ayelet Shai, Omry Koren

**Affiliations:** 1 Azrieli Faculty of Medicine, Bar-Ilan University, Safed, Israel; 2 Department of Oncology, Galilee Medical Center, Nahariya, Israel; 3 Department of Mathematics, Bar-Ilan University, Ramat Gan, Israel; 4 The Blavatnik School of Computer Science, Tel Aviv University, Tel Aviv, Israel; 5 Department of Clinical Microbiology and Immunology, Sackler Faculty of Medicine, Tel Aviv University, Tel Aviv, Israel; 6 Santa Fe Institute, Santa Fe, NM USA

**Keywords:** Microbiome, Weight gain, Adjuvant chemotherapy, Cancer, Germ-free mice

## Abstract

**Background:**

Adjuvant chemotherapy induces weight gain, glucose intolerance, and hypertension in about a third of women. The mechanisms underlying these events have not been defined. This study assessed the association between the microbiome and weight gain in patients treated with adjuvant chemotherapy for breast and gynecological cancers.

**Methods:**

Patients were recruited before starting adjuvant therapy. Weight and height were measured before treatment and 4–6 weeks after treatment completion. Weight gain was defined as an increase of 3% or more in body weight. A stool sample was collected before treatment, and 16S rRNA gene sequencing was performed. Data regarding oncological therapy, menopausal status, and antibiotic use was prospectively collected. Patients were excluded if they were treated by antibiotics during the study. Fecal transplant experiments from patients were conducted using Swiss Webster germ-free mice.

**Results:**

Thirty-three patients were recruited; of them, 9 gained 3.5–10.6% of baseline weight. The pretreatment microbiome of women who gained weight following treatment was significantly different in diversity and taxonomy from that of control women. Fecal microbiota transplantation from pretreatment samples of patients that gained weight induced metabolic changes in germ-free mice compared to mice transplanted with pretreatment fecal samples from the control women.

**Conclusion:**

The microbiome composition is predictive of weight gain following adjuvant chemotherapy and induces adverse metabolic changes in germ-free mice, suggesting it contributes to adverse metabolic changes seen in patients. Confirmation of these results in a larger patient cohort is warranted.

## Background

A complex link exists between obesity and cancer in women. Obesity and metabolic syndrome are associated with a higher prevalence of postmenopausal breast cancer, colon cancer, endometrial cancer, and ovarian cancer and with a higher risk of recurrence in patients that are treated for early-stage disease [1].

Adjuvant chemotherapy is given to patients after surgery for cancer, aiming to increase the proportion of patients that are cured. Adjuvant chemotherapy induces weight gain in about a third of women treated for breast and gynecological malignancies [2, 3]. Increase in body fat mass and serum lipids, increased blood pressure, and an increase in inflammatory markers have also been described following adjuvant chemotherapy in women [4]. A recent meta-analysis of trials testing adjuvant therapy in breast cancer patients concluded that weight gain after diagnosis had a negative impact on outcomes in pre-, peri-, and early postmenopausal patients [5]. Weight gain during chemotherapy was associated with reduced survival in prospective studies [6, 7]. Weight gain and the associated metabolic changes increase the risk of cardiovascular morbidity and other common and debilitating conditions and have a negative impact on patients’ quality of life. The mechanisms mediating the effect of adjuvant chemotherapy on weight and metabolism are not understood, limiting our ability to develop preventive measures and to predict which patients are at a higher risk.

The microbiome composition is distinctly different between obese and lean people and has been widely studied both in humans and in animal models. Obesity is characterized by low bacterial richness (alpha diversity) and high between-individual (beta) diversity [8, 9]. Furthermore, microbial changes in obesity are associated with inflammation, insulin resistance, and adiposity [10]. Mice that were transplanted with microbiota from obese individuals develop increased body mass and adiposity compared with mice that were transplanted with microbiota from lean individuals [8], suggesting that the microbiome is not only affected by diet but also actively induces obesity.

Previous studies looked at changes in the microbiome in patients receiving chemotherapy [11]. These studies showed that chemotherapy alters the microbiome and suggested an association between the microbiome and acute chemotherapy toxicities, mainly in the gastrointestinal system [12–14]. None of these studies looked at weight and late metabolic effects of chemotherapy.

The aim of the current study was to understand the interplay between the intestinal microbiome and weight gain in women treated with adjuvant chemotherapy for breast cancer and gynecological malignancies, and test whether the pretreatment microbiome in women who will gain weight following chemotherapy has obesogenic characteristics.

## Methods

### Patients and samples

Patients aged 18–75 years with breast, ovarian, or endometrial cancer scheduled for adjuvant chemotherapy were eligible for the study. Breast cancer patients scheduled for neoadjuvant chemotherapy were also eligible. Patients with inflammatory bowel disease and patients treated with probiotics were excluded. Patients provided a stool sample prior to the 1st chemotherapy cycle; samples were frozen immediately and delivered on dry ice to the Azrieli Faculty of Medicine, Bar-Ilan University, Safed, Israel. Weight and height were measured before the 1st chemotherapy cycle and 4–6 weeks after treatment was completed. Use of antibiotics in the prior 3 months was recorded at baseline, during chemotherapy, and at the last visit. Patients were excluded from the analysis if they used antibiotics during chemotherapy. Since prophylactic antibiotics before cancer surgery is standard of care, patients treated with antibiotics before trial entry were allowed. Patients referred for pelvic radiotherapy and those that experienced cancer progression were withdrawn from the study. Patients were entered to the “weight gain” group if their weight increased by 3% or more during treatment.

### Bacterial DNA extraction, amplification, and sequencing

DNA was extracted from women and mice fecal samples, using the Invitrogen Purelink Microbiome DNA extraction kit (Invitrogen, Carlsbad, CA) according to the manufacturer’s instructions, following a bead-beating step (BioSpec, Bartlesville, OK) for 2 min. Extracted DNA was used for PCR amplification of the variable V4 region of the 16S rRNA gene by using the 515F (AATGATACGGCGACCACCGAGATCTACACGCT) barcoded and 806R (TATGGTAATTGTGTGYCAGCMGCCGCGGTAA) primers. A reaction containing a final concentration of 0.04% of each primer and 0.5% of PrimeSTAR Max DNA Polymerase (Takara-Clontech, Shiga, Japan) in 50 μl total volume was used. PCR reactions were carried out by 35 cycles of denaturation (95 °C), annealing (55 °C), and extension (72 °C), with final elongation at 72 °C. PCR products were purified using AMPure XP magnetic beads (Beckman Coulter, Brea, CA) and quantified using Quant-iT PicoGreen dsDNA quantitation kit (Invitrogen, Carlsbad, CA). Samples were then pooled at equal amounts, loaded on 2% agarose E-Gel (Invitrogen, Carlsbad, CA), purified, and sent for sequencing using the Illumina MiSeq platform (Genomic center, Azrieli Faculty of Medicine, BIU, Israel).

### Mouse experiments

For fecal microbiota transplantation experiments, we chose patients that were not treated with antibiotics (*n* = 12) and 2 more were chosen randomly from those that received preoperative antibiotics in order to increase sample size. Pretreatment fecal samples from women who gained weight (*n* = 6) after treatment or not (*n* = 8) were resuspended in 1 ml sterile PBS under anaerobic conditions, vortexed, and debris allowed to settle. Administration to recipient germ-free Swiss Webster mice (8–10 weeks old) was performed by oral gavage using 200 μl of the supernatant. The microbiota-recipient mice were housed separately under SPF conditions (1 animal per cage), maintained on a 12-h light/dark cycle, and fed autoclaved food, with free access to water. Fecal pellets and blood samples were taken on days 0, 14, and 28. The animal study was compiled with the ARRIVE guidelines and approved by the Bar-Ilan ethics committee (ethics approval number 41-05-2018).

### Intraperitoneal glucose tolerance test

On days 14 and 28, intraperitoneal glucose tolerance test (IPGTT) was done on mice from both groups: control (*n* = 8) and weight gain (*n* = 6). The mice were fasted overnight for approximately 16 h by transferring them to clean cages with no food or feces. Subsequently, the mice were given a 20% glucose solution by an IP injection in a volume of 10 μl/1 g body weight. Blood glucose was measured from the tail at 0, 15, 30, 60, 90, and 120 min after the glucose challenge (Contour blood glucose meter, Bayer). At time points 0, 30, and 60 min, blood samples were collected (around 60 μl) using a fresh capillary tube coated with EDTA solution. Blood samples were immediately placed on ice. Tubes were then centrifuged at 1500*g* for 20 min at 4 °C. Plasma was transferred to a clean tube and stored at − 30 °C for further analysis. Finally, on day 28, the mice were sacrificed after 8 h fasting under CO_2_ and blood was taken from the heart.

### Insulin and lipocalin-2 analysis

Mouse blood was separated by centrifugation (1500*g* for 20 min at 4 °C), and the plasma stored at − 80 °C. Ten microliters from each plasma sample was taken for metabolic hormone (insulin) measurement, and 2 μl from each plasma sample was taken for lipocalin-2 measurement. The multiplex adipokine panel (MADKMAG-71 K, Merck Millipore) was used according to the manufacturer’s instructions to measure levels of insulin. The multiplex kidney injury panel (MKI2MAG-94 K, Merck Millipore) was used according to the manufacturer’s instructions to measure levels of lipocalin-2. The results were read using a Bio-Plex MAGPIX reader and analyzed with the Bio-Plex manager 6.1 software (Bio-Rad).

### Lipid analysis

On day 28, the mice were fasted for 8 h before sacrificed under CO_2_ and blood was taken from the heart for lipid content. Mouse blood was separated by centrifugation (1500*g* for 20 min at 4 °C), and the plasma stored at − 80 °C. Plasma levels of triglycerides, cholesterol total, HDL, and LDL were measured using the Abbott Architect clinical chemistry analyzer at the Galilee Medical Center clinical laboratory.

### Microbiome analysis

FASTQ data was processed and analyzed using Quantitative Insights Into Microbial Ecology 2 (QIIME2) pipeline version 2019.4 [15]. Single-end sequences were first demultiplexed using the q2-demux plugin. In order to improve taxonomic resolution, reads were denoised and clustered using DADA2 via q2-dada2 [16]. Mafft [17] and fasttree2 [18] were used for alignment and phylogeny construction for all amplicon sequence variants (ASVs) using q2-alignment and q2-phylogeny plugins, respectively. Taxonomy classification was done using q2-feature-classifier [19], while final feature sequences were aligned against Greengenes database with 99% confidence [20]. In order to avoid any possible contamination, the feature table was filtered via q2-feature-table. First, features that were annotated as mitochondria and chloroplast were filtered. Next, features which were not found in 20% of each sample group were removed, for both women and mice feature tables.

The analysis, for both women and mice, was performed on rarefied tables with > 9300 reads per sample. Alpha diversity was calculated using the Faith’s Phylogenetic Diversity [21] measure, referring for bacterial richness within the sample, while significant differences in bacterial richness between the groups were generated using the Kruskal-Wallis test. Beta diversity was analyzed using weighted (quantitative) and unweighted (qualitative) UniFrac [22] distances. Significance was determined using permutational multivariate analysis of variance (PERMANOVA) test, as implanted in QIIME2 with the default of 999 permutations, both weighted and unweighted UniFrac.

Significant differences in bacterial abundance were identified using Linear Discriminant Analysis (LDA) of the effect Size (LEfSe), with an LDA score higher than 2.0 and α values of 0.05 [23].

### Statistical analysis

Mouse body weights were normalized according to day 0, the first day of the experiment, in order to calculate changes between gain weight and control transplanted mouse groups over time. Differences in weight gain fold change, glucose levels, lipocalin-2 levels, and lipid content (measured by triglycerides, HDL, and LDL) were assessed using unpaired one-tailed *t* test. Food consumption was calculated using unpaired nonparametric *t* test. All data represent as mean ± SEM.

### Machine learning and clustering

The beta diversity of each pair of samples was used as a distance metric, and a single link hierarchical clustering was applied to predict groups of samples. A leave one out approach was then used to classify each sample based on all other samples with a *K* = 2 KNN classifier. The precision of the classifier was defined as the total accuracy over the test. Similarly, the alpha diversity was defined as a score for each sample, and a ROC curve was computed over the same groups using the alpha diversity score. The area under curve (AUC) was computed for the ROC curve, and the accuracy at the maximal accuracy cutoff was computed. Note that there was no division to train and test, since the score was predefined and not directly trained on the labels.

### Metagenome analysis

To infer metagenomes, meaning the microbial genes present in each sample, from the 16S samples, we used PICRUSt2 [24]. PICRUSt2 provides proportions of KEGG categories within the samples, and those categories were in turn used to look for differentially abundant functions between the weight gain and control groups (KEGG ortholog and pathway levels, Mann-Whitney test, FDR corrected, alpha = 0.05).

Moreover, we used FishTaco [25], both as an alternative approach for assembling the functional profiles and assessing functional differences between weight gain and control groups, as well as for identifying the taxonomic origin of such differences, if any.

## Results

Thirty-three patients were recruited, 28 with breast cancer and 5 with gynecological malignancies. Ten patients gained 3.5–10.6% of baseline weight, and most of them gained more than 5%. No significant differences were observed in food consumption based on food diaries taken from 15 patients, before, during, and after chemotherapy treatment. Five parameters were measured: calories, protein, fat, carbohydrate, sugar, and fiber intake (Fig. S1). Patient and chemotherapy characteristics are summarized in Table 1. Twelve patients were withdrawn from the analysis: 7 patients used antibiotics during chemotherapy, 3 had missing data, 1 was recruited but did not commence chemotherapy, and 1 was referred for pelvic radiotherapy and was withdrawn from the study per protocol. Mean age, menopausal status, baseline BMI, site of cancer, and treatment regimens were not significantly different between the groups that did and did not gain weight (Table 2 and Fig. S2).

**Table 1:** Patients and treatment characteristics (only patients who were included in analysis)

Patient number	Age group range	Site of cancer	Baseline BMI range	Menopause	Chemotherapy regimen	Percentage of weight change	Fecal microbiota transfer
**Control**							
2	50–60	Breast	> 30	Post	Adriamycin + cyclophosphamide, paclitaxel	− 2.8	No
3	60–70	Breast	25–30	Post	Adriamycin + cyclophosphamide, paclitaxel	− 0.7	No
6	70–75	Breast	25–30	Post	Adriamycin + cyclophosphamide, paclitaxel	− 7.8	No
7	60–70	Breast	20–25	Post	Adriamycin + cyclophosphamide, paclitaxel	0.0	Yes
11	60–70	Breast	25–30	Post	Paclitaxel	1.6	Yes
15	50–60	Endometrium	> 30	Post	Paclitaxel + carboplatin	1.2	No
16	60–70	Breast*	> 30	Post	Adriamycin + cyclophosphamide, paclitaxel	− 4.9	Yes
18	60–70	Breast*	20–25	Post	Adriamycin + cyclophosphamide, paclitaxel + carboplatin	− 2.0	Yes
19	60–70	Endometrium	25–30	Post	Paclitaxel + carboplatin	0.1	Yes
26	40–50	Breast	20–25	Post	Adriamycin + cyclophosphamide, paclitaxel	− 2.0	Yes
29	30–40	Breast*	< 20	Pre	Adriamycin + cyclophosphamide, paclitaxel	− 5.2	Yes
32	40–50	Breast	> 30	Pre	Docetaxel + carboplatin	− 1.5	No
33	50–60	Breast	20–25	Pre	Docetaxel	− 4.1	No
**Mean**	**56.77**		**27.65**			**− 1.9**	
**Weight gain**							
1	50–60	Endometrium	25–30	Post	Paclitaxel + carboplatin	10.2	Yes
4	40–50	Breast	> 30	Pre	Paclitaxel	6.1	Yes
5	60–70	Breast	25–30	Post	Adriamycin + cyclophosphamide, paclitaxel	3.5	Yes
20	60–70	Breast	25–30	Post	Paclitaxel	5.5	Yes
21	50–60	Breast	> 30	Post	Adriamycin + cyclophosphamide, paclitaxel	11.5	Yes
22	40–50	Breast	20–25	Pre	Adriamycin + cyclophosphamide, paclitaxel	7.2	Yes
24	50–60	Ovary	> 30	Post	Paclitaxel + carboplatin	10.7	Yes
30	60–70	Breast	20–25	Post	Adriamycin + cyclophosphamide	3.5	No
**Mean**	**55.50**		**28.05**			**7.3**	

*Neoadjuvant therapy for breast cancer

**Table 2:** The association of the different variables tested with weight gain after adjuvant chemotherapy. We used a Pearson correlation for continuous factors. For discrete factors, we used an ANOVA analysis

Variable	*p* value
Age	0.5379
Menopause	0.6745
Baseline BMI	0.1987
Site of cancer	0.2475
Chemotherapy regimen	0.1085
Microbiome beta diversity	0.012
Microbiome alpha diversity	0.01

**Fig. 1:**
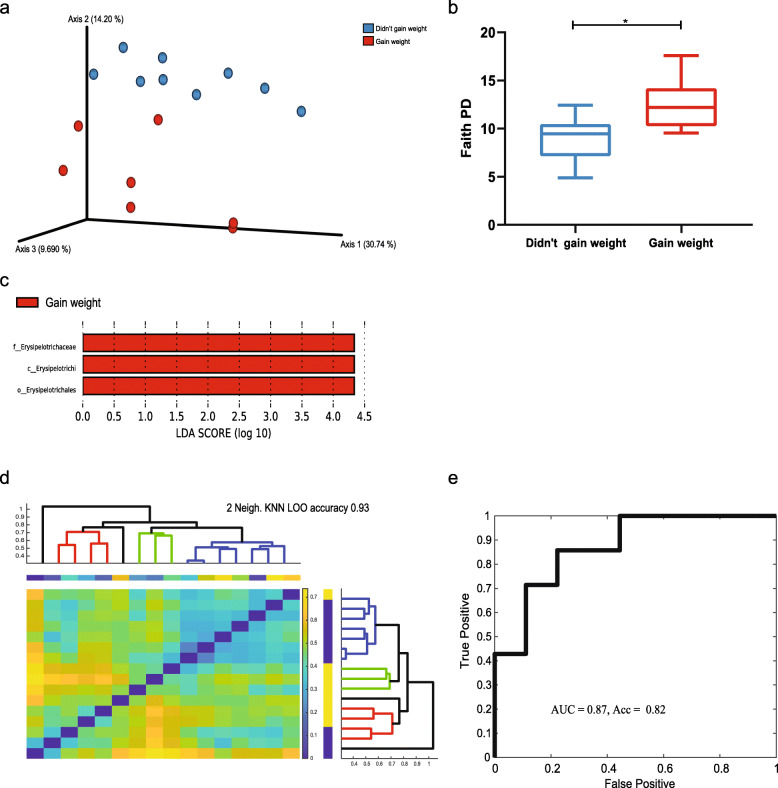
The pretreatment gut microbiome of women who will gain weight following chemotherapy is different from the gut microbiome of women who will not gain weights. 16S rRNA sequencing was performed to characterize bacterial changes. **a** Principal coordinates analysis based on unweighted UniFrac distance matrix between women that will (red) and will not (blue) gain weight following chemotherapy treatment (*p* value = 0.012). **b** Alpha diversity comparison based on phylogenetic diversity (*p* value = 0.01). **c** Linear Discriminant Analysis (LDA) of the effect Size (LEfSe). **d** A single link hierarchical clustering based on an unweighted UniFrac distance matrix. *K*-nearest neighbor (KNN) classifier was used for classification. The colors in the heatmap represent beta diversity values. The blue and yellow bars on the right represent control and weight gain, respectively. The bar on the top represents different women. The lines in the dendrogram are colored based on the clusters. Black lines in the dendrogram represent samples different from the three main clusters. **e** The area under curve (AUC) for the ROC curve based on alpha diversity analysis

The microbiome analysis was based on 16 fecal samples from women before chemotherapy, out of which 9 women did not gain weight following treatment (control group), and 7 women gained weight following treatment (weight gain group). All women did not receive antibiotics during chemotherapy. Beta diversity based on unweighted UniFrac distances revealed significant differences between the two groups (Fig. 1a, *p* value = 0.012). Significant differences were also observed in alpha diversity using Faith’s PD, pointing to a more diverse pretreatment microbiome in the weight gain group (Fig. 1b, *p* value = 0.01). LEfSe analysis identified a higher relative abundance of members of the family Erysipelotrichaceae (and at the class and order level too) in the pretreatment microbiomes of women that gained weight after chemotherapy (Fig. 1c). Based on the gut microbiome composition, we tested whether beta and alpha diversity could be used to predict which of the patients will gain weight after chemotherapy treatment. Samples were divided into those that gained weight and those that did not gain weight (Fig. 1d). A single link hierarchical clustering (Fig. 1d) was performed on the beta diversity as a distance. This demonstrated that almost all the nearest neighbors of samples were from the same group. Indeed, a *K*-nearest neighbor (KNN) classifier (with *K* = 2) produced an accuracy of over 87%. Similarly, the alpha diversity could be used as classifier by itself, with a slightly lower accuracy (82%) (Fig. 1e).

### The contribution of the intestinal microbiome to metabolic changes

**Fig. 2:**
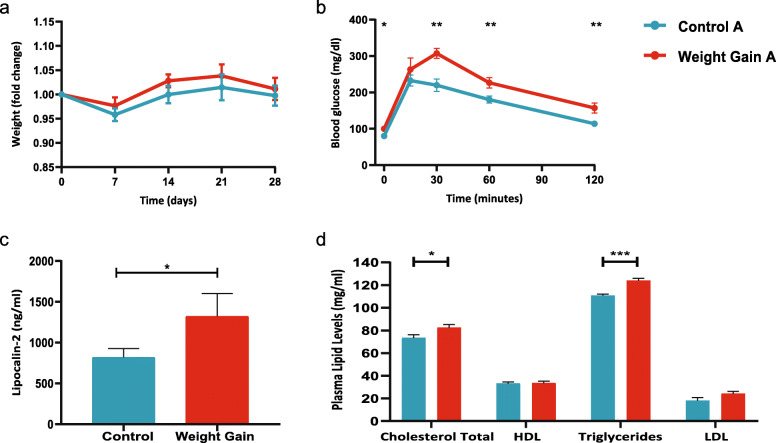
FMT using pretreatment samples from women that will gain weight after chemotherapy to GF mice induces significant changes compared to FMT from women that will not gain weight (control). **a** Weight (fold change) of mice receiving FMT from women that will gain weight and patients that will not gain weight after chemotherapy treatment. **b** Blood glucose levels based on intraperitoneal glucose tolerance test (IPGTT) 28 days after FMT. **c** Lipocalin-2 levels 28 days after FMT. **d** Lipid levels in plasma 28 days after FMT

In order to test the role of the intestinal microbiome in inducing weight and metabolic changes in women undergoing adjuvant chemotherapy, we performed fecal microbiota transplantation (FMT) experiments. Mice transplanted with microbiome from patients (pretreatment) that gained weight were compared to mice transplanted with microbiome from patients that did not gain weight after chemotherapy treatment. No significant differences were observed in weight between the groups for 28 days (Fig. 2a). However, at day 28, mice receiving FMT from pretreatment samples from the weight gain group had significantly higher glucose levels 0, 30, 60, and 120 min following intraperitoneal glucose injection compared to mice that received FMT from pretreatment samples of the control group (Fig. 2b). The same was true for lipocalin-2 levels (Fig. 2c, *p* value = 0.04) and total cholesterol and triglycerides (Fig. 2d, *p* value = 0.02 and *p* value = 0.0001, respectively), which were higher in mice receiving FMT from women who gained weight after therapy.

**Fig. 3:**
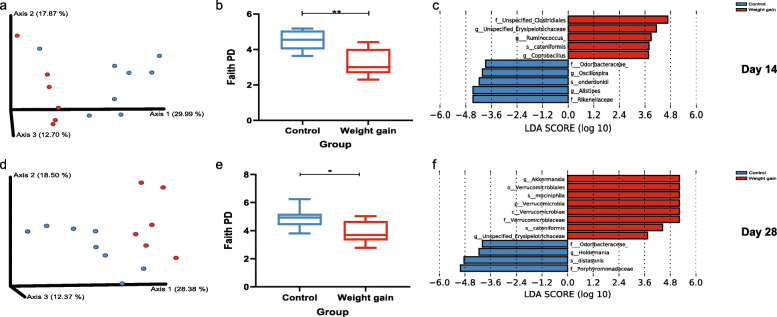
FMT using pretreatment samples from women that will gain weight after chemotherapy to GF mice induces significant microbial changes compared to women that will not gain weight (control). **a**, **d** PCoA of unweighted UniFrac distances at **a** 14 (*p* value = 0.003) and **d** 28 days (*p* value = 0.001) post-FMT. **b**, **e** Alpha diversity at **b** 14 (*p* value = 0.009) and **e** 28 days (*p* value 0.03) post-FMT. **c**, **f** Cladogram generated using Linear Discriminant Analysis (LDA) of the effect Size (LEfSe) **c** 14 and **f** 28 days after FMT. (*p < 0.05, **p < 0.01, ***p < 0.001, and ****p < 0.0001; data represent the mean ± SEM of at least 6 samples in each group)

The mice microbiome analysis was done at days 14 and 28 of the experiment. Beta diversity based on unweighted UniFrac distances (Fig. 3a, d) was significantly different at both time points (*p* value = 0.003 and *p* value = 0.001, respectively). When comparing alpha diversity using Faith’s Phylogenetic Diversity (Fig. 3b, e), significant differences were also observed at days 14 and 28 (*p* value = 0.009 and *p* value = 0.03, respectively). To tease out which bacteria differed between the experimental groups, we conducted a LEfSe analysis (Fig. 3c, f). On day 14, mice receiving FMT from the control group had higher relative abundance of the genera *Alistipes* and *Oscillospira* and of the bacterial families Odoribacteraceae and Rikenellaceaea. Mice receiving the pretreatment samples from the weight gain group had significantly higher levels of *Ruminococcus*, *Coprobacillus*, and an unclassified member of the Erysipelotrichaceae.

On day 28, LEfSe analysis identified the genus *Holdemania* and the bacterial families Odoribacteraceae (which was also higher at day 14) and Porphyromonadaceae as significantly higher in the control mice. In the mice transplanted with the pretreatment samples from the weight gain group, *Akkermansia* was overrepresented compared to the control group together with an unclassified member of the Erysipelotrichaceae (which was also overrepresented in this group on day 14). Comparative analysis of the functional profiles based on the outputs of both PICRUSt2 and FishTaco did not show a statistically significant difference between the weight gain and control groups.

## Discussion

Women treated with adjuvant chemotherapy are at risk for weight gain and other adverse metabolic consequences like hypertension, lipid metabolic changes, and chronic inflammation [4]. The reason for these changes is not clear, nor is the reason why some women are more prone than others to these adverse consequences. Previous studies have shown that different factors, such as physical activity and energy consumption, can play a role in weight gain during chemotherapy treatment [26, 27]. Those factors are also known to affect the gut microbiome composition [28]. However, in this study, we show for the first time that the pretreatment microbiome of patients that gained weight following adjuvant chemotherapy is different than the microbiome of patients that did not gain weight, and that fecal transplantation from patients that gained weight results in glucose intolerance, adverse lipid changes, and inflammatory changes in germ-free mice. These results suggest that the intestinal microbiome is mediating metabolic changes in women treated by chemotherapy in the adjuvant setting. Moreover, the pre-chemotherapy composition of the intestinal microbiome can predict which patients will gain weight following treatment.

A recent study by Carter et al. looked at the microbiome of women recovering from cancer and showed that microbiome diversity was associated with cardiorespiratory fitness in 37 women after breast cancer treatment [29]. However, their cohort was heterogeneous, and chemotherapy was not a part of adjuvant therapy in 30% of patients. The association of the microbiome with BMI or weight change was not analyzed; however, in line with our results, microbiome diversity was negatively associated with percentage of body fat.

The bacterial family of Erysipelotrichaceae, which was more abundant in pretreatment samples of women that gained weight following treatment, has been indicated in multiple studies to have a role in metabolic disorders [30–32]. One possible mechanism suggested has been the immunogenic properties of some of the members of this family which may lead to gut inflammation and to weight gain [30]. Members of this family were also overrepresented in the mouse experiment at 14 and 28 days in the mice who received FMT from the weight gain group, once again highlighting the potential contribution of this family to weight gain. It is also worth mentioning that members of this family have been linked to cholesterol and lipid levels which in our study were shown to be higher in the mice receiving FMT from the weight gain group [30, 33]. On the other hand, members of the family Odoribacteraceae persisted in the control mice. Bacteria in this family are known succinate consumers, and levels of succinate are lower in obesity [34], possibly explaining why these bacteria were significantly overrepresented in the control group.

To our knowledge, this is the first study looking at the microbiome and late consequences of adjuvant chemotherapy. Adjuvant chemotherapy is administered to patients in order to decrease risk for disease recurrence, and treatment decisions must balance its benefits with the risk of long-term toxicity. The microbiome is a modifiable risk factor for obesity [35, 36] and can serve both as a biomarker and a target for intervention.

Our cohort was well characterized, and data regarding oncological therapies and antibiotic use was systematically collected. We chose to include patients with breast, ovarian, and endometrial cancer and to exclude women with gastrointestinal and other cancer types since the chemotherapy regimens that are used in breast and gynecological malignancies are relatively similar. However, there are some limitations to the study. The size of our cohort does not allow firm conclusions and the results need to be verified in a larger patient cohort. We are currently enrolling additional patients and plan to study additional metabolic changes such as lipids, glucose, and blood pressure and their association with the microbiome in each type of cancer separately and with specific chemotherapy drugs. Additional mice experiments that include chemotherapy administration are also planned.

## Conclusions

In this study, the composition of the intestinal microbiome and its diversity were associated with weight gain following adjuvant chemotherapy in women treated for breast and gynecological malignancies. Mice FMT experiments suggest that the microbiome mediates adverse metabolic effects of chemotherapy. Further research of the predictive value of the microbiome, as well as its mechanistic contribution to weight and metabolic changes following chemotherapy, is warranted.

## Supplementary information

**Additional file 1: Figure S1.** Dietary intake from patient that gained and those that did not gain weight after chemotherapy, based on food diaries. **Figure S2.** Bar graph presenting the association between weight gain and different clinical factors (LIST).

## Data Availability

Data was submitted to EBI–ERP123368.

## Abbreviations

FMT: Fecal microbiota transplantation
ASVs: Amplicon sequence variants
QIIME: Quantitative Insights Into Microbial Ecology
GF: Germ-free
HDL: High-density lipoprotein
LDL: Low-density lipoprotein
SEM: Standard error of the mean
