# Supplementary material for: The intestinal microbiome, weight, and metabolic changes in women treated by adjuvant chemotherapy for breast and gynecological malignancies
Source: BMC Med. 2020 Oct 21;18:281. doi: 10.1186/s12916-020-01751-2 (PMC7576808; doi:10.1186/s12916-020-01751-2)
Supplement: Supplementary file 1 — Additional file 1: Figure S1. Dietary intake from patient that gained and those that did not gain weight after chemotherapy, based on food diaries. Figure S2. Bar graph presenting the association between weight gain and different clinical factors (LIST). [file 12916_2020_1751_MOESM1_ESM.docx]

Supplementary Materials

**The intestinal microbiome, weight and metabolic changes in women treated by adjuvant chemotherapy for breast and gynecological malignancies.**

Atara Uzan-Yulzari^1,^*, Maya Morr^2,^*, Hala Tareef Nabwani^1,^*, Oren Ziv^1^, Dafna Magid Neriya^3^, Ran Armoni^4^, Efrat Muller^4^, Anca Leibovici^2^, Elhanan Borenstein^4,5,6^, Yoram Louzoun^3^, Ayelet Shai^1,2,^*, Omry Koren^1,$,^*


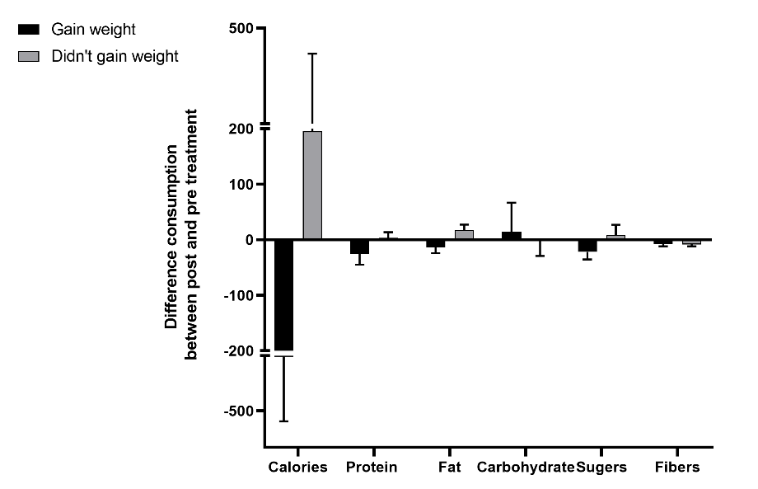


**Fig S1. Dietary intake from patient that gained and those that did not gain weight after chemotherapy, based on food diaries.**

Food diaries were filled out by the initial 15 patient. Differences in patient's food consumption, normalized per day, post and pretreatment, were calculated using Unpaired, nonparametric ttest. No significant differences were detected in all parameters; Calories, protein, fat, carbohydrate, sugars and fibers.


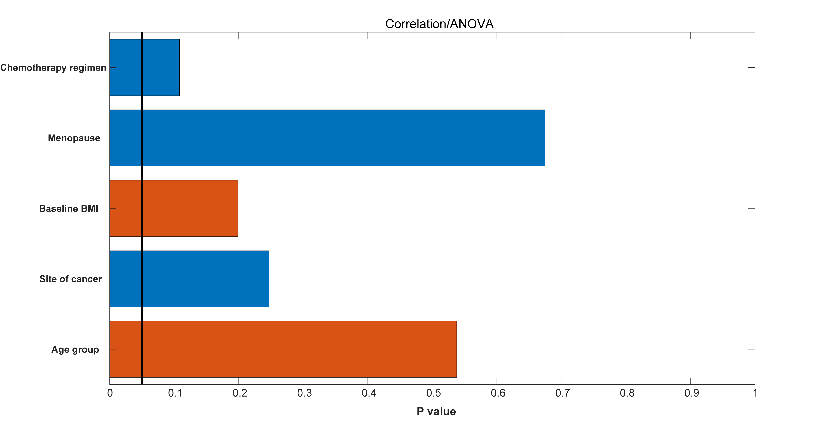


**Fig S2. Bar graph presenting the association between weight gain and different clinical factors (LIST)**.

Pearson correlation was used to examine continuous factors (baseline BMI and age group), for discrete factors (chemotherapy regimen, menopause and site of cancer) ANOVA analysis was used. No significant associations were detected. Statistics are presented in table 2.
